# Epigenetic therapies targeting histone lysine methylation: complex mechanisms and clinical challenges

**DOI:** 10.1172/JCI183391

**Published:** 2024-10-15

**Authors:** Sarah Gold, Ali Shilatifard

**Affiliations:** Department of Biochemistry and Molecular Genetics and Simpson Querrey Institute for Epigenetics, Northwestern University Feinberg School of Medicine, Chicago, Illinois, USA.

## Abstract

As epigenetic therapies continue to gain ground as potential treatment strategies for cancer and other diseases, compounds that target histone lysine methylation and the enzyme complexes represent a major frontier for therapeutic development. Clinically viable therapies targeting the activities of histone lysine methyltransferases (HKMT) and demethylases (HKDMs) have only recently begun to emerge following FDA approval of the EZH2 inhibitor tazemetostat in 2020 and remain limited to compounds targeting the well-studied SET domain–containing HKMTs and their opposing HKDMs. These include the H3K27 methyltransferases EZH2/EZH1, the singular H3K79 methyltransferase DOT1L, and the H3K4 methyltransferase MLL1/COMPASS as well as H3K9 and H3K36 methyltransferases. They additionally include the H3K4/9-preferential demethylase LSD1 and the H3K4-, H3K27-, and H3K36-preferential KDM5, KDM6, and KDM2 demethylase subfamilies, respectively. This Review discusses the results of recent clinical and preclinical studies relevant to all of these existing and potential therapies. It provides an update on advancements in therapeutic development, as well as more basic molecular understanding, within the past 5 years approximately. It also offers a perspective on histone lysine methylation that departs from the long-predominant “histone code” metaphor, emphasizing complex-disrupting inhibitors and proximity-based approaches rather than catalytic domain inhibitors in the outlook for future therapeutic development.

## Introduction

Epigenetic therapies that target histone lysine methylation (HKme) represent a major frontier for the treatment of cancer and other diseases. Broadly defined, epigenetic therapies are compounds that target the chromatin-associated protein factors, including chromatin-modifying enzymes, that coordinate the relationship between the genome and the transcriptome. In the prevailing epigenetic metaphor known as the “histone code” hypothesis (1, 2), these factors are said to “write” (deposit), “erase” (remove), or “read” (bind to) “epigenetic marks” (DNA methylation and posttranslational histone modifications).

Epigenetic therapies were initially conceptualized as a means to “turn on” the expression of tumor suppressor genes in cancer cells. The first epigenetic therapies were hypomethylating agents (HMAs), covalent inhibitors of DNA methyltransferase enzymes developed to counteract the aberrant patterns of transcriptionally repressive DNA methylation observed in hematological malignancies. HMAs became clinically viable in 2004, when 5-azacytidine gained initial FDA approval for the treatment of myelodysplastic syndrome (MDS). Low-dose HMAs are now the standard frontline treatment for high-risk MDS and chemotherapy-ineligible acute myeloid leukemia (AML), and they are also frequently used to prime or potentiate response to other therapies (3). A second wave of development focused on inhibition of histone deacetylase (HDAC) enzymes as an alternative route to “turn on” tumor suppressor genes via the unchecked accumulation of histone lysine acetylation (HKac), a transcriptionally permissive modification. Following initial FDA approval of vorinostat for the treatment of cutaneous T cell lymphoma in 2006, several other HDAC inhibitors have since gained FDA approval for the treatment of various hematological malignancies and other diseases (thoroughly reviewed in ref. 4). HKac factor-targeting compounds have also been developed to impede or “turn off” oncogenic or proliferation-sustaining gene expression in cancer. These include inhibitors of histone acetyltransferase enzymes, such as p300/CBP, and compounds that target chromatin-associated effector proteins that “recognize” HKac, such as BET family proteins. They have yet to meet with clinical success, as we discuss in a recent review (5). In contrast, epigenetic therapies that target HKme now have a champion in the EZH2 inhibitor tazemetostat, which gained expedited FDA approval for the treatment of certain patients with epithelial sarcoma or follicular lymphoma (FL) in 2020.

**Figure 1:**
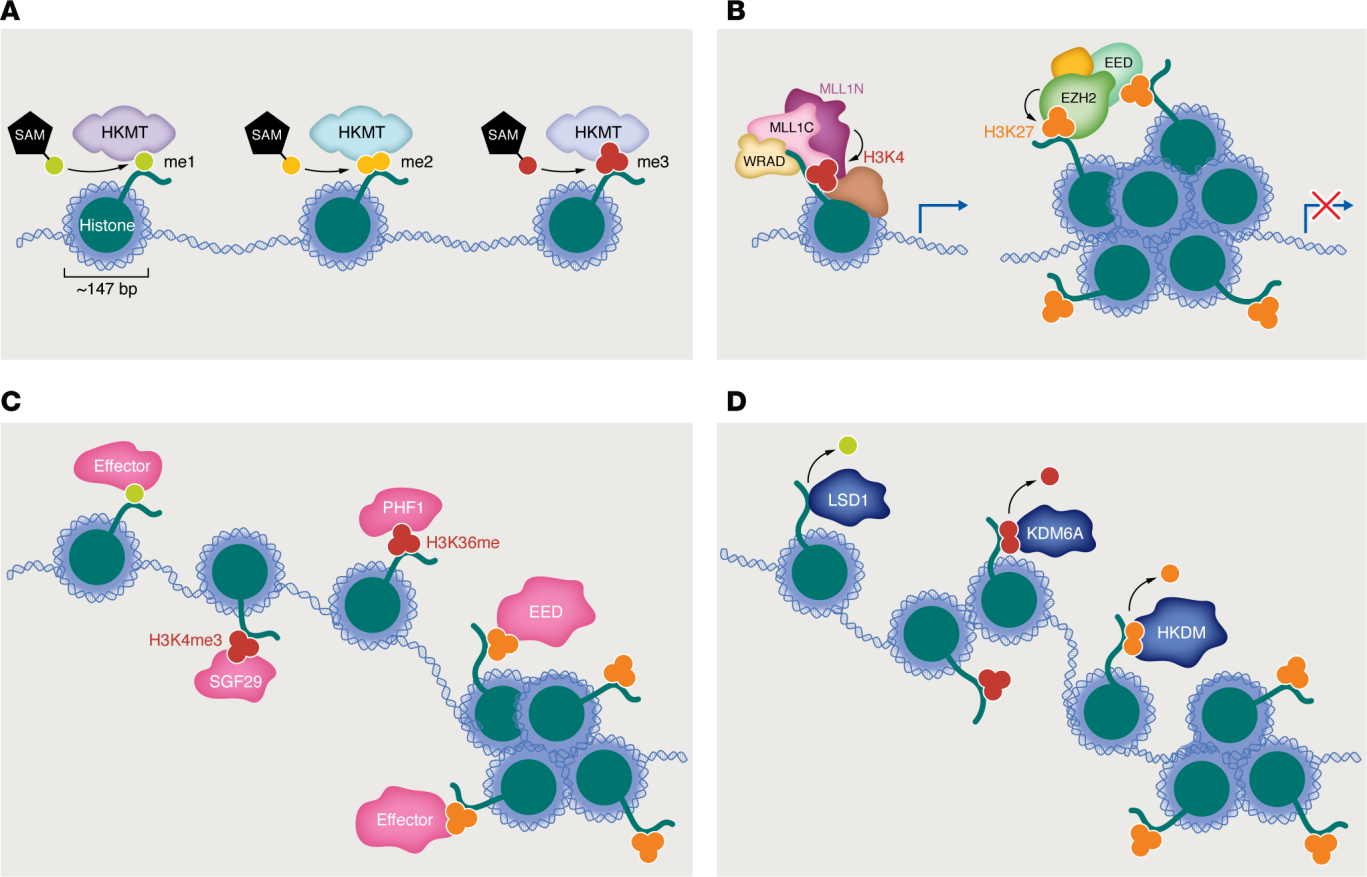
An overview of histone lysine methylation. (**A**) Histones are the protein subunits of nucleosomes, essential chromatin structures in which approximately 147 bp of genomic DNA is wound around a histone core. Lysine residues within the globular core or the unstructured tails of histone proteins can be mono, di, or trimethylated (me1, -2, or -3) by the catalytic activity of histone lysine methyltransferase (HKMT) enzymes. (**B**) MLL1/COMPASS (left) and other HKMT in the trithorax/COMPASS family deposit histone 3 lysine 4 (H3K4) methylation “marks” that are considered to act as positive regulators of gene expression, while EZH2 and other HKMT in the polycomb family deposit H3K27 methylation that is considered to facilitate transcriptional repression. Subunits of the core WRAD module (present in all COMPASS complexes) are shown with labels in Figure 3A. (**C**) Methylated histone lysines can recruit a plethora of different methylation, residue, and context-specific effector proteins to regulate gene expression. SGF29, PHF1, and EED are examples of histone methylation-binding effector proteins. SGF29 recruits a version of the PRC2 complex by binding H3K36me3 via its Tudor domain. PHF1 recruits the SAGA transcriptional coactivator complex by binding H3K4me3 via its Tudor domain. The effector function of the PRC2 complex subunit EED, which binds to H3K27me3 via its WD40 repeat domain, is illustrated in Figure 2A. (**D**) Methylation is removed by the activity of lysine demethylase (HKDM) enzymes.

HKme is a type of posttranslational histone modification that is generally thought to regulate the dynamics of transcription and gene expression but also includes diverse modifications of mostly unknown function. It is critical to emphasize that “modification” simultaneously refers to two distinct concepts: a deposited “mark” *and* the dynamic process of its deposition. (We have previously discussed whether a definite function can be ascribed to an epigenetic mark; see refs. 6–8). Let us briefly reintroduce histones as the protein subunits of nucleosomes, essential chromatin structures in which approximately 147 bp of genomic DNA is wound around a histone core (Figure 1A). Lysine residues within the globular core or the unstructured tails of histone proteins can be mono-, di-, or trimethylated (me1, -2, or -3) by the catalytic activity of histone lysine methyltransferase (HKMT) enzymes (Figure 1A). The activity of HKMTs in the trithorax/COMPASS and polycomb families, which have in common catalytic Su(var)3-9, enhancer-of-zeste, trithorax (SET) domains, are particularly well-studied. The histone 3 lysine 4 (H3K4) methylation deposited by HKMTs in the trithorax/COMPASS family is considered to act as a positive regulator of gene expression, and the H3K27 methylation deposited by HKMTs in the polycomb family is considered to facilitate transcriptional repression (Figure 1B) (9, 10). Unless or until it is removed or countered by the activity of lysine demethylase (HKDM) enzymes (Figure 1D), HKme can recruit a plethora of different effector proteins to regulate gene expression (Figure 1C). Which specific effectors are recruited depends on the methylation state of the histone lysine, its position, and the context of nearby modifications. HKme occurs alongside HKac (and other acylation, e.g., succinylation), in some cases even on a single lysine residue (11), and lysines are only one of several modifiable histone residues. As a result, effectors may selectively “recognize” (bind with relatively high affinity or avidity) specific modifications or combinations of modifications on a single nucleosome.

We will attempt to discuss all epigenetic therapies currently developed to target components of the cellular machinery that deposit or remove HKme. As many such therapies have been previously (and thoroughly) reviewed (12), this Review is intended to serve as an update covering the results of recent clinical and preclinical studies within the last approximately 5 years. Notably, the scope of development in this area remains limited to compounds targeting SET domain–containing HKMTs and their opposing HKDMs. We will discuss therapies targeting the H3K27 methyltransferases EZH1 and EZH2 first, as these are most numerous and clinically advanced following the approval of tazemetostat (Table 1 and Table 2). We will discuss therapies targeting the singular H3K9 methyltransferase DOT1L and the H3K4 methyltransferase MLL1 next, as these have been or are now in clinical stages of development for the treatment of various leukemias (Table 1 and Table 2). We will then discuss newly developed therapies targeting H3K36 and H3K9 methyltransferases, which remain in preclinical stages (Table 2). We will also discuss the therapies targeting LSD1, some of which have been clinically evaluated (Table 1), as well as potential therapies targeting other HKDMs, particularly in combination therapies (Table 2). We will conclude with a brief discussion of the outlook for future development, given the complexity and redundancy of the mechanisms involved.

## H3K27 methyltransferase-targeting therapies

**Figure 2:**
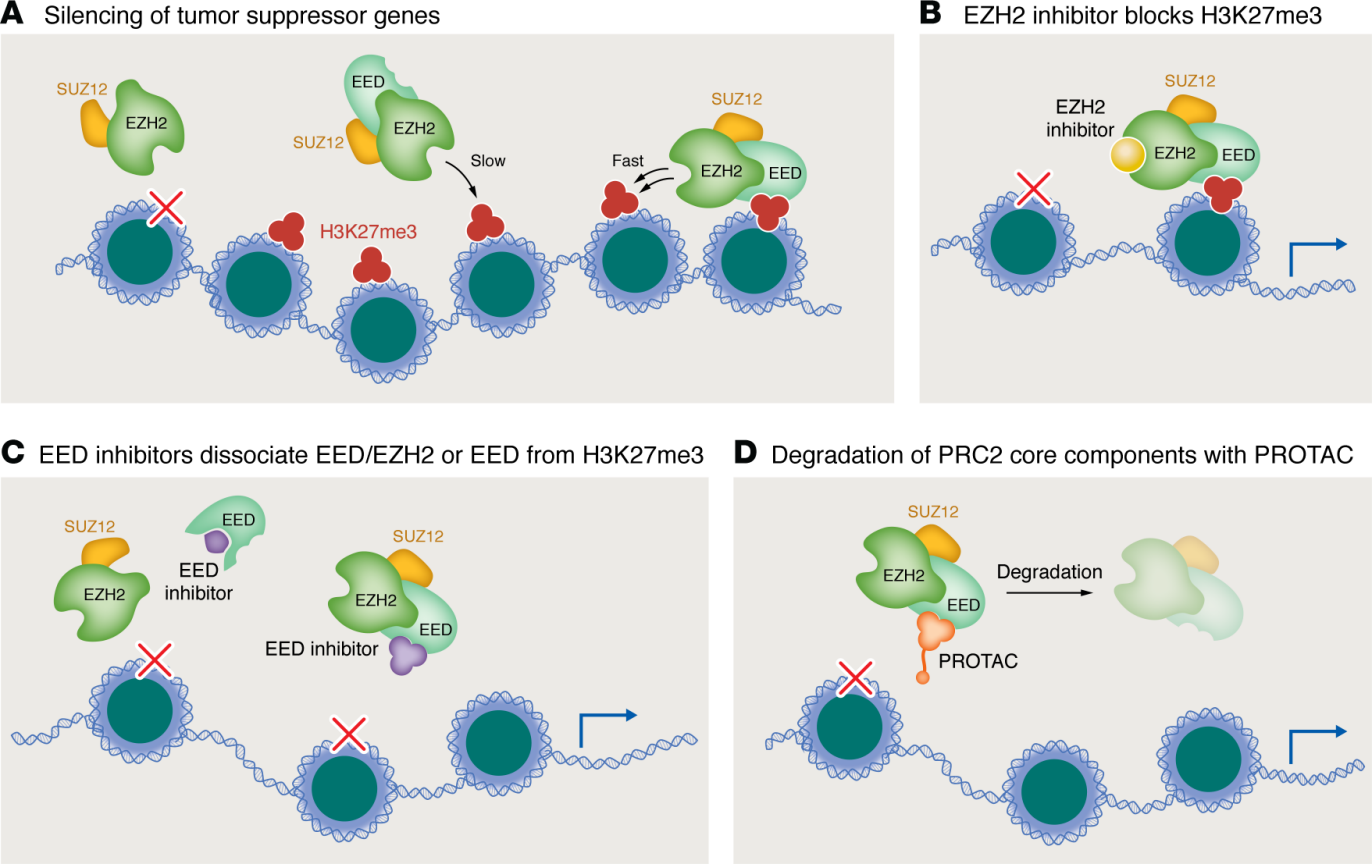
An emerging inhibitor class indirectly targets EZH2 activity via the PRC2 subunit EED. The PRC2 complex catalytic subunit EZH2 is a target for cancer therapy because its H3K27me3-depositing activity silences the expression of tumor suppressor genes. However, new targets are needed to overcome acquired resistance to existing EZH2 inhibitors. (**A**) Baseline EZH2 activity is enabled by interaction with the PRC2 subunit EED, which can also bind to H3K27me3, stimulating 10- to 20-fold greater EZH2 activity toward H3K27 on neighboring nucleosomes to facilitate “spreading” of this repressive modification. (**B**) EZH2 inhibitors such as the FDA-approved compound tazemetostat directly target the catalytic activity of EZH2 via the EZH2 SET domain. (**C**) EZH2 activity can be indirectly targeted via small molecules that disrupt EED’s EZH2-interacting pocket or its H3K27me3-binding pocket. (**D**) EED-targeting molecules can be adapted into proteolysis-targeting chimeras (PROTACs) by fusion via a linker peptide to a ligand of an E3 ubiquitin ligase, which targets EED for proteasomal degradation and may also lead to degradation of EZH2 and other PRC2 subunits.

The polycomb complex PRC2 deposits transcriptionally repressive H3K27 methylation via the catalytic activity of its methyltransferase subunit, a tissue- and context-specific role that may be played by either EZH1 or EZH2 (Figure 2A). This H3K27 methyltransferase activity is counteracted by the activity of the KDM6 subfamily demethylases KDM6A (UTX) and KDM6B (JMJD3). PRC2 activity is also regulated by histone H2A ubiquitination, which is deposited by the monoubiquitinase activity of the polycomb complex PRC1 and removed by the deubiquitinase activity of the BAP1 complex (9).

Several inhibitors have been developed to target the H3K27 methyltransferase activity of PRC2. One class of inhibitors directly targets the catalytic SET domains of EZH2 and/or EZH1 (Figure 2B). The most prominent example is the S-adenosyl-methionine–competitive (SAM-competitive), EZH2-selective inhibitor tazemetostat, which gained expedited FDA approval for the treatment of certain patients with epithelial sarcoma or FL in 2020. Gain-of-function mutations that constitutively activate EZH2 (especially missense mutations of tyrosine 641) are highly prevalent in non–Hodgkin lymphoma–type (NHL-type) B cell lymphomas of germinal center origin (13), and tazemetostat was specifically indicated for adult patients with FL with an identified EZH2 mutation based on clinical trial results. However, a recent reanalysis of the phase 2 E7438-6000-101 trial concluded that the apparently superior efficacy observed in patients with FL with EZH2 mutation versus patients with wild-type EZH2 might be due to clinical disparities in the baseline characteristics of these two groups (14), suggesting that tazemetostat may be indicated for patients with FL regardless of their EZH2 mutation status. Tazemetostat was recently evaluated in combination with the PD-L1 blocker atezolizumab for the treatment of relapsed/refractory (R/R) diffuse large B cell lymphoma (DLBCL), with tolerable safety but only modest antitumor activity reported (objective response rate [ORR] 16%) (15). Biomarker analysis in the study identified EZH2 mutations (gain of function) in only four of 28 evaluable patients, but three of these had either partial (PR) or complete responses (CRs), while patients without EZH2 mutations achieved stable disease (25%) as a best response. Further evaluation of EZH2 inhibition in combination with PD-L1 blockade may be warranted in patients with DLBCL or FL, ideally including patients with both activating and inactivating mutations, given that the EZH2 mutation-specific efficacy of tazemetostat is unclear.

Broader applicability of PRC2 inhibition to the treatment of other cancers is supported by the general prevalence of mutations in other chromatin-associated proteins that perturb the balance of activities regulating H3K27me3, including components of the SWI/SNF chromatin remodeling complex and the BAP1 H2A deubiquitinase complex (16). Tazemetostat was recently evaluated for the treatment of pediatric tumors (primarily rhabdoid tumors) with EZH2 mutation or loss of the SWI/SNF subunits SMARCB1 or SMARCA4 (17). Though the overall response rate was low, PR was achieved for the single patient with SMARCA4 loss, and disease was stabilized for an average of fifteen months for four other patients, all with SMARCB1 loss (17). Tazemetostat was also recently evaluated for the treatment of patients with R/R malignant pleural mesothelioma with BAP1 inactivation (assessed as absence of nuclear BAP1) (18). Disease was controlled in 54% of patients at 12 weeks. A significant decrease in tumor-infiltrating B cells, particularly B cells within tumor stroma (no direct tumor cell contact), was observed after tazemetostat treatment in eight of the ten patients for which paired tumor biopsies were available (immunohistochemistry and RNA-Seq–based population estimate results were apparently obtained but not reported for other tumor-infiltrating immune cell types) (18). Because the effect of intratumoral B cells on antitumor immunity and treatment outcomes likely depends on their distinct phenotypes and intratumoral “neighborhood” contexts (e.g., tertiary lymphoid structures), as well as tumor type (19), the ultimate effect of apparent tazemetostat-induced intratumoral B cell depletion in malignant pleural mesothelioma, as well as the relevance of this finding to other cancers, remain unclear.

In contrast to countering BAP1 inactivation via EZH2 inhibition, producing BAP1 inactivation may also be a therapeutic goal in specific disease contexts. For example, our group recently demonstrated that frameshift mutations affecting the BAP1 complex subunit ASXL1 (which are frequently observed in myeloid malignancies) create truncated gain-of-function ASXL1 mutants that stabilize the BAP1 complex and increase its recruitment to chromatin (20). We identified compounds that could selectively inhibit BAP1 deubiquitinase activity using an enzymatic activity assay approach, then demonstrated preclinical antitumor efficacy of BAP1 inhibition using these compounds in cell line and xenograft models of myeloma with ASXL1 frameshift mutations (20). A cryo-EM-resolved structure of nucleosome-bound BAP1 and the deubiquitinase activation domain of ASXL1 (preserved in truncated ASXL1 proteins) was recently published (21), and we look forward to future development of structure-guided, BAP1 complex-disrupting inhibitors.

EZH2 inhibition could potentially be used to increase tumor immunogenicity and/or improve responses to immunotherapies. Investigating how a combination of the MEK inhibitor trametinib and the CDK4/6 inhibitor palbociclib (T/P) could induce an inflammatory senescence-associated secretory phenotype in lung adenocarcinoma but not pancreatic ductal adenocarcinoma (PDAC), a recent study found that elevated EZH2 activity in pancreatic tumors suppressed expression of proinflammatory genes in response to T/P treatment. Either EZH2 shRNA knockdown or tazemetostat treatment rescued proinflammatory gene expression, increasing surveillance by NK and CD4^+^ T cells (22). Interestingly, while low-dose tazemetostat (in combination with T/P) increased infiltrating CD8^+^ T cells, high-dose tazemetostat (in combination with T/P) was conversely found to decrease infiltrating CD8^+^ T cells in this mouse model of KRAS-driven PDAC (22). Our group recently evaluated the effect of EZH2 inhibition on bladder tumors using a fully immunocompetent, carcinogen-induced mouse model of muscle-invasive bladder cancer, finding that targeting EZH2 not only inhibited tumor progression but also induced immune responses including upregulated MHC II, increased CD3^+^ tumor-infiltrating lymphocytes (NK, CD4^+^, CD8^+^), and tertiary lymphoid structures (23). Moreover, reproducing this model in adaptive immune-deficient *Rag1*-knockout mice proved that adaptive immunocompetency is required for the antitumoral activity of EZH2 inhibition. In fact, EZH2 inhibition *potentiated* tumor progression in immunodeficient mice (23).

Combinations with other epigenetic therapies might extend the applicability of EZH2 inhibition. Synergistic cytotoxicity was recently reported for the EZH2 inhibitor GSK126 in combination with the HMA decitabine in hepatocellular carcinoma cell lines, in which GSK126 alone had little cytotoxic effect (24). A synergistic effect was also reported for tazemetostat in combination with the BET inhibitor JQ1 in pediatric atypical teratoid/rhabdoid tumors characterized by SMARCB1 loss (25). This synergy may not be attributable to effects of JQ1 on H3K27ac-dependent gene regulatory processes, because a recent study using pan-H3K27R mutant mESCs (in which all *H3* alleles are Cas9-edited to replace lysine residues with arginine) demonstrated that H3K27ac is dispensable for the reactivation of transcriptional activity and gene expression upon loss of H3K27me (26) (also suggesting limited synergy for potential combinations of EZH2 and HAT inhibitors). However, another study recently demonstrated that H3K36me2 deposited by the methyltransferase NSD1 is required for transcriptional activation upon EZH2 inhibition, with NSD1 loss conferring resistance to EZH2 inhibition in SMARCB1-deficient rhabdoid tumor cell lines that could be rescued by further inhibition of the H3K36 demethylase KDM2A (27). This finding suggests the possible therapeutic combination of EZH2 and KDM2A inhibition in rhabdoid tumors with EZH2 mutation or SWI/SNF deficiency.

Though selective EZH2-targeting strategies have been the primary subject of research focus and regulatory approvals, the possibility of compensatory EZH1 activation may call this approach into question. The dual EZH1/EZH2 inhibitor valemetostat was recently evaluated in adult patients with R/R T cell leukemia/lymphoma (ATL) (28), which arises due to human T-lymphotropic virus type 1 (HTLV-1) infection. Promising efficacy (48% ORR, 20% CR, and 28% PR) and a tolerable safety profile were reported, and valemetostat gained approval for the treatment of R/R ATL patients in Japan in 2022. Distinct from ATL, HTLV-1 infection-associated myelopathy (HAM) involves hyperimmune responses, including proliferation of HTLV-1^+^ CD8^+^ T cells. A recent study demonstrated that dual EZH1/2 inhibition suppressed proliferation of PBMCs from patients with HAM in vitro, similar to the prednisolone standard of care. While prednisolone primarily suppressed CD4^+^ T cell proliferation, EZH1/2 inhibition suppressed proliferation of both CD4^+^ and CD8^+^ T cells and reduced HTLV-1 proviral load in PBMCs (29). HTLV-1 is not yet endemic in Europe or North America, but valemetostat is now in clinical trials for the treatment of BCL in Europe and for NHL (ATL, BCL, and peripheral T cell lymphoma) in joint studies in both Japan and the United States (30). Recent preclinical evidence also supports dual EZH1/2 inhibition over EZH2-selective inhibition for the treatment of malignant rhabdoid tumors with SMARCB1 loss (31).

Drug resistance due to somatic mutations is a major challenge for therapies that target the EZH2 SET domain. For example, a recent paper identified a recurrent somatic mutation in lymphoma, W113C, that affects a SET-activating loop to impart gain-of-function in H3K27 methyltransferase activity (32). Importantly, this mutation is reported to confer resistance to EZH2 SET domain inhibition but not to inhibitors that target EZH1/2 activity indirectly by targeting the PRC2 complex member EED (32). EED has two targetable “pockets” (Figure 2C): one that interacts with EZH2 to enable its baseline activity, and another that binds H3K27me3 to stimulate 10- to 20-fold greater PRC2 activity toward H3K27 of neighboring nucleosomes (Figure 2A) (33). The clinical candidate EED inhibitor MAK683, which targets this H3K27me3-binding pocket, has undergone extensive preclinical investigation (34). Recently reported inhibitors targeting the EED/EZH2 interaction (35) may also yield clinical candidates soon. Finally, EED-targeting inhibitors can be adapted into proteolysis-targeting chimeras (PROTACs), a proximity-based approach that targets EED for proteasomal degradation via fusion of the EED-targeting compound to a ligand of an E3 ubiquitin ligase via a linker peptide (Figure 2D). A recent study demonstrated that targeting EED in this way led to the degradation not only of EED but also of EZH2 and an additional PRC2 component, SUZ12 (36). Importantly, another recent study demonstrated that EZH2-targeting PROTACs (in which tazemetostat is linked to an E3 ligand) acted similarly, leading to the degradation not only of EZH2 but also of EED and SUZ12 (37).

The most common treatment-related adverse events for existing EZH2-targeting therapies are cytopenias (thrombocytopenia, anemia, neutropenia). These are generally manageable, and recent reports indicate that thrombocytopenia in particular might be reduced via combination with low-dose HMA (38–40). However, it remains critical to investigate on-target but adverse effects, especially as these therapies are potentially applied to a wider range of indications. For example, EZH2 loss or loss of function has consistently been shown to result in resistance to a variety of chemotherapies in hematological malignancies (41–43). There may be additional cardiovascular risks: a recent study demonstrated that EZH2 inhibition via GSK126 treatment increased vascular stiffness in a mouse model (markedly more pronounced in young compared with middle-aged mice) and in primary human aortic smooth muscle cells from young donors, concomitant with increased expression of matrix metalloproteinase 2 (MMP2) from an H3K27me3-depleted and H3K27ac-enriched promoter (44).

## H3K79 and H3K4 methyltransferase-targeting therapies

MLL-rearranged (MLLr) leukemias are common forms of infant and childhood leukemia that also occur secondary to chemotherapy in adult patients. In MLLr leukemias, the N-terminal of the COMPASS family H3K4 methyltransferase MLL1 (*KMT2A*) is fused with the C-terminal of any one of many fusion partners, including subunits of the DOT1L complex (DOTCOM) and the super elongation complex (SEC) (45, 46). DOT1L is the only known H3K79 methyltransferase, and many inhibitors have been developed to target its catalytic activity, including SAM-competitive inhibitors and inhibitors that bind a pocket adjacent to the SAM binding site (these and other strategies are thoroughly reviewed in ref. 47.) The SAM-competitive inhibitor pinometostat is the only DOT1L inhibitor that has been evaluated in clinical trials; a favorable safety profile but modest clinical benefit supports its further evaluation in combination with other therapies (48). However, given the catalytic-independent functions we have demonstrated for DOT1L (49) and the pinometostat resistance mechanisms identified by single-cell CRISPR tiling of the DOT1L gene (50), targeted DOT1L degradation or DOTCOM disruption strategies might be required. A recent study identified compounds that disrupt either DOTCOM or SEC by targeting the AF9/ENL AHD domain, which mediates recruitment of elongation machinery via interaction with the AHD domain of AF4/AFF4, and demonstrated preclinical antitumor efficacy for these compounds in cell line and animal models of MLLr leukemia (51). As previously reviewed, DOTCOM or SEC activity could also be disrupted in rearranged leukemias via recently developed therapies targeting AF9/ENL YEATS domains (5, 52, 53).

**Figure 3:**
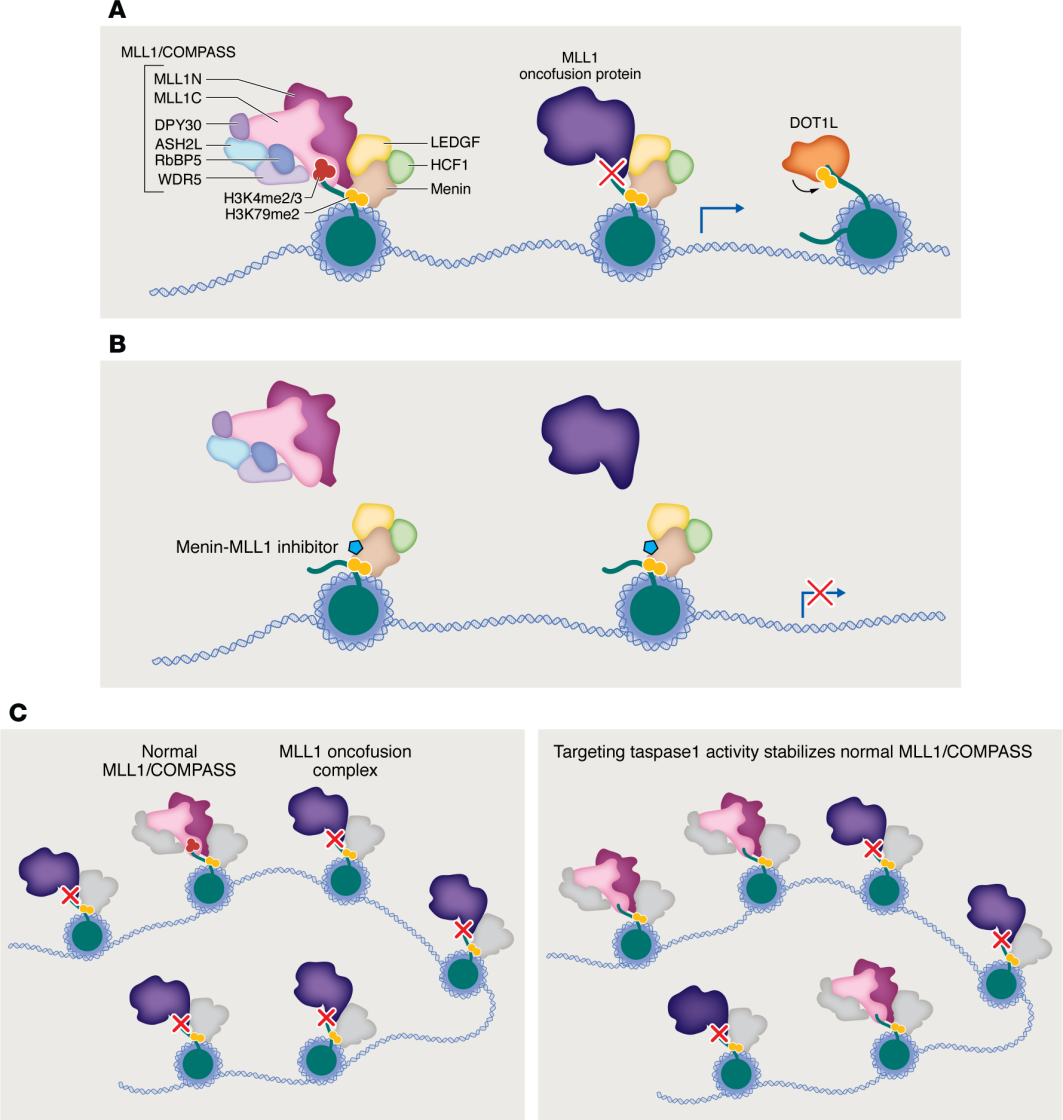
Noncatalytic inhibitors evict MLL1 and its oncofusion partners from chromatin by disrupting the menin-MLL1 interface. (**A**) The COMPASS methyltransferase MLL1 (left) epigenetically modifies histone 3 lysine 4 with dimethylation and trimethylation (H3K4me2/3) via the catalytic activity of its SET domain. MLL1/COMPASS contains MLL1N and MLL1C subunits, products of MLL1 cleavage by taspase1. Menin tethers the MLL1/COMPASS complex to chromatin via its interaction with LEDGF. It was recently reported that menin also binds chromatin via recognition of histone 3 lysine 79 dimethylation (H3K79me2), a modification that is exclusively deposited by the methyltransferase DOT1L, which is a frequent oncofusion partner of MLL1 in leukemias driven by 11q23 translocations. The SET domain–containing C-terminal region of MLL1 is lost in MLL1 oncofusion proteins. (**B**) Disruption-based inhibitors target an N-terminal domain of MLL1 that binds menin. Inhibitors that disrupt the menin-MLL1 interface can evict both MLL1/COMPASS and MLL1 oncofusion proteins from chromatin. (**C**) MLL1 oncofusion proteins, which lack the taspase1 cleavage site present in normal MLL1, form more stable chromatin-associated complexes than normal MLL1/COMPASS (left). Stabilizing MLL1/COMPASS (e.g., by targeting taspase1 activity) is a potential strategy for rebalancing chromatin occupancy in favor of normal MLL1/COMPASS (right).

Noncatalytic, disruption-based inhibitor strategies have already been used to target MLL1 fusion proteins, which lack a targetable SET domain in MLL1 due to loss of its C-terminal region upon chromosomal rearrangement affecting *KMT2A* (Figure 3A). Disruption-based inhibitors instead target an N-terminal MLL1 domain that binds the scaffold protein menin, which tethers MLL1 (or MLL2) to chromatin via its interaction with LEDGF (54). Inhibitors that disrupt the menin-MLL1 interaction can therefore evict MLL1 and its fusion partners (e.g., DOTCOM) from chromatin (Figure 3B). Recent preclinical results confirm that they can also evict the MLL1-colocalized mutant NPM1 proteins found in *NMP1*-mutant (NPM1c) AML, the most common adult AML subtype (55). Recently published results of the first-in-human phase I study of the menin-MLL1 inhibitor revumenib for the treatment of MLLr or NPM1c R/R acute leukemia indicate a favorable safety profile and early evidence of clinical benefit, with a CR rate of 20% and ORR of 53% (56). Some patients in this study developed resistance to revumenib, which a subsequent mutation analysis determined was conferred by mutations at the menin-MLL interface (57). However, synergistic combinations that maximize effectiveness within the therapeutic window for menin inhibition could potentially contravene the development of resistance. For example, a recent preclinical study demonstrating that menin-MLL1 inhibition downregulated BCL2 and CDK6 and that CRISPR knockout or dTAG depletion of menin increased sensitivity to the BCL2 inhibitor venetoclax or the CDK6 inhibitor abemaciclib and further demonstrated synergistic effects of revumenib and related compounds in combination with venetoclax or abemaciclib in xenograft models derived from patients with MLLr or NPM1c AML (58). Potential use of the unrelated menin-MLL1 inhibitor ziftomenib in combination with either the BCL2 inhibitor venetoclax or the BET inhibitor OTX015 is also supported by synergistic cytotoxicity, as recently demonstrated in cells derived from patients with MLLr or NPM1c AML(59). Another recent preclinical study demonstrated synergistic effects of menin-MLL inhibition in combination with various inhibitors of the tyrosine kinase receptor FLT3, including the FDA-approved AML therapies quizartinib and gilteritinib, in AML cells with *FLT3* mutations (found in 10% of MLL1r and 60% of NPM1c AML patients) (60). It was recently reported that menin “reads” (selectively binds to) H3K79me2 via its finger domains, allowing for coincident interaction via its palm and N-terminal domains with MLL1 and LEDGF (61); small-molecule disruptors of this interaction could therefore be used in place of or in combination with menin-MLL1 inhibitors in MLLr leukemias or NPM1c AML. Because H3K79me2 is deposited exclusively by DOT1L, this major finding also suggests a possible feed-forward loop in leukemias driven by MLL1-DOT1L fusion, which may support combined menin-MLL1 and DOT1L inhibitor therapy.

Although MLL1 fusion proteins lack the C-terminal region required for interaction with the COMPASS subunit WDR5, inhibitors that disrupt the specific interaction between MLL1 and the WIN site of WDR5 can be used to disrupt the remaining catalytic activity of the normal MLL1 allele. In vivo antitumor activity has been demonstrated for recently developed selective inhibitors of the MLL1-WDR5 interaction (62, 63). Importantly, a recent study comparing WDR5 WIN site inhibition to acute WDR5 depletion via an auxin-inducible degron demonstrated that WIN site inhibition disrupts only a subset of the WDR5 function as assessed by transcriptional downregulation upon rapid depletion of WDR5 (64), suggesting limited off-target effects for MLL1-WDR5 interaction disruptors on the function of other COMPASS complexes. However, WIN site mutations are also predicted to cause resistance to disruptors of MLL1-WDR5 interaction in MLLr leukemia (65). Somewhat paradoxically, a potential alternative to disruption of the remaining normal MLL1/COMPASS in MLLr leukemias may actually be *stabilization* of the normal MLL1/COMPASS. Our group previously demonstrated that loss of the MLL1 taspase1 cleavage site in MLL1 oncofusion proteins stabilized these chimeras on chromatin relative to normal MLL1/COMPASS and that inhibition of the taspase1 cleavage-facilitating phosphorylation activity of casein kinase II stabilized normal MLL1/COMPASS, shifting the balance of chromatin occupancy to favor normal versus chimeric MLL1(Figure 3C) (66).

In addition to its central role in MLLr and NPM1c leukemias, MLL1 also plays a role in leukemias driven by chromosomal rearrangement affecting the nucleoporin protein NUP98. MLL1 physically interacts with NUP98 fusion proteins, including fusions with the H3K36 methyltransferase NSD1 and the H3K4 demethylase KDM5A (JARID1A) (67). A recent study reporting MLL1 and menin as molecular dependencies in NUP98-rearranged AML demonstrated that the menin inhibitor VTP50469 (closely related to revumenib) extended survival in patient-derived xenograft models of NUP98-KDM5A and NUP98-NSD1 AML (68). VTP50469-extended survival was extremely long-lasting in the NUP98-NSD1 model (over 250 days after the treatment period), but survival duration was shorter for the responding NUP98A-KDM5A model, and an additional, heavily mutated NUP98A-KDM5A model did not respond to VTP50469 (68). Because a recent study identified EZH2 as a driver of NUP98r AML (69), the combination of menin and EZH2 inhibitors could be a rational therapeutic approach in this context. Recent work from our group identified compensatory MLL1 activity as a driver of metabolic rewiring in the absence of MLL3/4 function, suggesting that MLL1/COMPASS may also be a targetable molecular dependency in cancers with mutations affecting MLL3, MLL4, or their shared COMPASS partner, the H3K27 demethylase KDM6A (70).

## H3K9 and H3K36 methyltransferase-targeting therapies

The SUV family of methyltransferases deposits euchromatin-associated and transcriptionally repressive H3K9 methylation marks. SUV39H1 was actually the first mammalian HKMT to be identified, quickly followed by its homolog SUV39H2, in 2000 (71). Neither of these pericentric euchromatin-maintaining H3K9 methyltransferases appears to have been a target of interest for therapeutic development, likely due to their critical roles in the preservation of genomic stability and their abundant nonhistone targets (71). The remaining members of the SUV family have all drawn interest as targets for therapeutic development due to their elevated expression levels in various cancers, but this interest has not yet been translated into clinically viable therapies. The H3K9 methyltransferase SETDB1 has been reported to play a tumor suppressor role by limiting the expression of genes targeted by MLL1 fusion proteins (72), contraindicating SETDB1 inhibition in the treatment of MLLr leukemias. However, experimental evidence supports roles for SETDB1 in tumorigenesis (including metastatic progression via epithelial-mesenchymal transition) and in immune regulation (73). Recent reports indicate that SETDB1 suppresses stress-related expression of endogenous retrovirus, contributing to immune checkpoint inhibitor resistance and the development of an immunosuppressive tumor microenvironment (74), and that the endogenous retrovirus expression in SETDB1 deficiency has a radiosensitizing effect (75). These results suggest that SETDB1 inhibitors or degraders could be used to prime or potentiate chemo-, immuno-, or radiotherapies in appropriate contexts.

An enormous body of preclinical literature supports the therapeutic potential of inhibitors targeting G9A/GLP (EHMT2/EHMT1) in cancer and in a variety of other disease contexts, including inflammatory conditions and neurodegenerative disorders. Oddly enough, the S-adenosylhomocysteine inhibitor 3-deazaneplanocin A (DZNep), which had for many years been developed as an RNA methylation inhibitor and potential antiviral therapy, was only reported to act as an EZH2 inhibitor (specifically, a PRC2-depleting inhibitor of H3K27 but not H3K9 methylation) following identification of the first known G9A/GLP inhibitor, BIX-01294 (76, 77). DZNep and BIX-01294 together spurred further development of methyltransferase inhibitors, leading to the development of tazemetostat. Unfortunately, poor absorption, distribution, metabolism, excretion, toxicity (ADMET) properties have presented challenges for therapeutic development of G9A/GLP inhibitors thus far, which stalled after the quinazoline G9A/GLP inhibitor UNC-0642 was reported as an in vivo probe in 2013 (78). The most notable developments since have been dual inhibitors of both EZH2 and G9A/GLP. These include the substrate-competitive, dual EZH2 and G9A/GLP inhibitor HKMTI-1-005, which was demonstrated to induce differentiation in AML cells and induce responsiveness to all-*trans* retinoic acid (ATRA) treatment (79). We also note a trend of preclinical studies attempting to establish G9A/GLP inhibitors as potential therapies for addiction, substance abuse disorders, and other trauma-associated conditions, including anxiety and PTSD. However, because new reports reveal that existing quinazoline G9A/GLP inhibitors are also allosteric modulators capable of desensitizing the nicotinic acetylcholine receptor (nAChR), which is implicated in the pathology of all of these conditions (80, 81), potential G9A/GLP inhibitor-mediated epigenetic effects must be clearly distinguished from more direct effects on nAChR function in any future studies of this kind.

With the exception of the singular H3K36me3-depositing SETD2, which appears to unambiguously function as a tumor suppressor (82), the remaining H3K36 methyltransferases (NSD1, NSD2, NSD3, and ASH1L) are all implicated in or associated with various cancers. NSD2 (MMSET) and NSD3 are highly expressed in many cancers. NSD2 is especially prevalent in multiple myelomas arising from t(4;14) chromosomal translocation, which was associated with poor prognosis before the advent of proteasome inhibitor (bortezomib, carfilzomib, or ixazomib) therapy. NSD3 is located within an 8p11-p12 region that is amplified in approximately 10%–15% of breast cancers and approximately 20% of squamous cell lung cancers (LUSCs) (83). NSD1 and ASH1L are not highly expressed in cancer. However, NUP98:NSD1 fusions occur frequently in pediatric AML and are associated with poor prognosis (84), an NUP98:ASH1L fusion was recently identified by optical gene mapping in secondary AML (85), and ASH1L also appears to play a role in leukemia driven by MLL1:AF9 fusion (86). The SET domains of these H3K36 methyltransferases have been difficult to target due to obstruction of their active sites by autoinhibitory loops. Nevertheless, highly selective inhibitors targeting the SET domains of NSD1 (87), NSD2 and NSD3 (88, 89), and ASH1L (90) have all been recently reported. Moreover, inhibitors have also been developed to target the N-terminal PWWP H3K36me2 “reader” domains of NSD2 (91, 92) and NSD3 (93), and these PWWP-targeting compounds have now been adapted as binder moieties in PROTACs targeting NSD2 (94) and NSD3 (95, 96). Interestingly, a short NSD3 isoform (that lacks a SET domain but retains a H3K36me2-binding PWWP domain) was previously reported to play a role in MLLr leukemias by coupling the BET family protein BRD4 to the chromatin remodeler CHD8 (97). It was also recently demonstrated that LUSCs with high levels of NSD3 expression are highly sensitive to BET domain inhibition (83). Together, these results indicate a role for NSD3-BRD4 interaction and suggest therapeutic potential for combined NSD3 PWWP and BET domain inhibition (or degradation) in seemingly unrelated cancer contexts.

## Histone demethylase-targeting therapies

In addition to the methyltransferases that methylate histone lysine residues, the demethylase enzymes that remove these modifications (many of which participate in macromolecular complexes with methyltransferases and other chromatin modifiers) are also potential therapeutic targets. Development in this area has largely been limited to the demethylase LSD1 (KDM1A), which opposes the methyltransferase activities of MLL1 and SETDB1 by preferentially removing the mono- and di-methylation they deposit on H3K4 and H3K9, respectively. LSD1 was the first HKDM to be identified in 2004, followed by LSD2 in 2009. LSD1 and LSD2 are the only FAD-dependent HKDMs [the others are Fe(II), 2-oxoglutarate, and oxygen dependent]. Many naturally occurring flavones, alkaloids, and other compounds with monoamine oxidase inhibitor (MAOI) activity have been demonstrated or suggested to inhibit LSD1 (98). Pharmaceutical MAOI inhibit LSD1 activity via covalent interaction with FAD within the active site.

More selective covalent LSD1 inhibitors have so far been unsuccessful in clinical trials (these have been recently and thoroughly reviewed in ref. 99). Noncovalent, reversible LSD1 inhibitors have also been clinically evaluated. Phase I trials were terminated for the compound seclidemstat, which appears based on recent in vitro evidence to not effectively inhibit LSD1 (100); however, a rollover protocol allows patients to remain on this therapy, and another phase I trial will evaluate its combination with topotecan and cyclophosphamide for patients with Ewing sarcoma and other pediatric sarcomas (101). For the noncovalent LSD1 inhibitor pulrodemstat, results of a recent phase I dose-escalation (102) and dose-expansion (103) study for NHL and advanced solid tumors indicate that, despite only modest efficacy as a monotherapy, pulrodemstat offers a more favorable benefit-to-risk ratio versus covalent inhibitors, warranting its further evaluation in combination with other therapies. Most recently, noncovalent LSD1 inhibitors using a substrate-mimicking quinazoline scaffold have been evaluated as antiproliferative agents in AML, breast cancer, and rhabdomyosarcoma cell lines (104). Structurally, these quinazolines form a three-molecule “cap” via extensive ring stacking that blocks FAD access to the LSD1 active site (104).

The MAOI and nonselective LSD1 inhibitor tranylcypromine (TCP) was recently evaluated in combination with ATRA for the treatment of R/R AML and MDS, with an acceptable safety profile and evidence of clinical benefit including an ORR of 23.5%, where response was associated with a quiescent (hypoproliferative) CD34^+^ phenotype (105). ATRA induces cell differentiation and is extremely effective in combination with arsenic trioxide chemotherapy for the treatment of acute promyelocytic leukemia, a subtype of AML driven by PML:RARA fusion. However, ATRA is not effective in other AML types. Though far less effective than other LSD1 inhibitors as a cytotoxic agent (100), TCP was previously demonstrated to sensitize both APL and non-APL AML cells to differentiation-inducing ATRA treatment (106). Interestingly, genetic *LSD1* deletion has been demonstrated to induce ATRA sensitization, enhancer derepression, and cell differentiation independently of LSD1 catalytic activity, with structural evidence suggesting a mode of action in which TCP inhibits interaction of LSD1 with the SNAG domain-containing protein GFI1 (106), and transcriptional and genome-wide occupancy evidence suggesting that LSD1 loss not only affects its CoREST complex partners, but also allows for p300/CBP binding at enhancers (106, 107).

Apart from LSD1, the H3K4-preferential KDM5 subfamily has drawn the most interest for therapeutic targeting, in part because KDM5A is a fusion partner in NUP98r AML (64). KDM5A was recently reported to be required for cMYC-driven transcription in MM cells, and a KDM5-selective inhibitor in this same study limited growth of MM cell lines and patient samples, reduced tumor burden and improved survival in a disseminated tumor model, and inhibited tumor growth in a subcutaneous plasmacytoma model (108). KDM5B, however, has been identified as a tumor suppressor in MLLr AML (109) and NUP98r AML (69), and KDM5C has also recently identified as a tumor suppressor, with a particularly strong association between KDM5C expression and long-term progression-free survival in female patients with AML (110). Together, these results indicate the need for caution in use of KDM5 inhibitors unless they are strongly selective for KDM5A.

The H3K27-preferential KDM6 subfamily (KDM6A and KDM6B) should also be targeted with caution. The KDM6-selective inhibitor GSK-J4 has shown promising results in preclinical models (111) and was recently shown to radiosensitize DIPG cells, resulting in synergistic increase in survival upon combined GSK-J4 and radiation therapy in a xenograft mouse model (112). However, KDM6A appears to function as a tumor suppressor (113), and recent results implicate KDM6A loss in bladder cancer progression (114) and resistance to the CD38 blocker daratumumab in multiple myeloma (115), potentially contraindicating the general use of KDM6A/B inhibitors.

On a brighter note, inhibition of the H3K36 demethylase KDM2A was recently demonstrated to rescue resistance to EZH2 inhibition conferred by loss of the H3K36 methyltransferase NSD1 in SMARCB1-deficient rhabdoid tumor cell lines, as discussed above (27). This finding suggests the possible therapeutic combination of EZH2 and KDM2A inhibitors in rhabdoid tumors with EZH2 mutation or SWI/SNF deficiency. Another recent report indicates that KDM2A inhibition may also be applicable to the treatment of a broader set of cancers that rely on alternative telomere maintenance, for which KDM2A is a critical factor (116). To our knowledge, KDM2A inhibitors have not yet been developed as potential therapeutic candidates.

## Conclusion

Assessing the current status of these therapies overall, our major conclusion is that there are already a plethora of candidates with the potential to follow the EZH2 inhibitor tazemetostat into clinical application. However, we believe that the HKme-targeting epigenetic therapies most likely to be successful in the clinic are not those that directly target the catalytic activity of a HKMT or HKDM (6, 7).

Instead, we expect to see successful therapeutic application of compounds with several other novel modes of action. These include compounds that target the noncatalytic domains of HKMTs (such as the PWWP methylation “reader” domains of NSD2 and -3), the noncatalytic subunits of HKMT-containing complexes (such as the PRC2 subunit EED) or other chromatin modifiers that regulate HKMT activity (such as the BAP1 subunit ASXL1), compounds that disrupt protein-protein interactions within HKMT complexes (such as the interaction between MLL1 and menin) or within complexes that regulate HKMT activity (such as the BAP1 ubiquitinase complex), and proximity-induced degraders that eliminate all activities, both catalytic and noncatalytic, of a given HKMT or HKDM (such as anticipated PROTACs targeting LSD1). Compounds that target the Tudor or other methylation “reader” domains of effector proteins (Figure 1C) represent another exciting therapeutic horizon (117).

As discussed previously (7), the long-predominant “histone code” (1, 2) metaphor may limit our ability to consider HKme as a spatially constraining process that colocalizes specific methylation-depositing and -removing machinery (along with the other chromatin modifiers within their larger macromolecular complexes) at a given loci. HKMTs are also increasingly understood to modify or otherwise interact with transcription factors, such as p53 and BRD4. We hope that future therapeutic development in this area will take fully into account the physical complexity of the mechanisms by which HKMTs, other chromatin modifiers, and transcription factors together facilitate gene and context specificity in the transcriptional regulation of gene expression.

## Figures and Tables

**Table 2:**
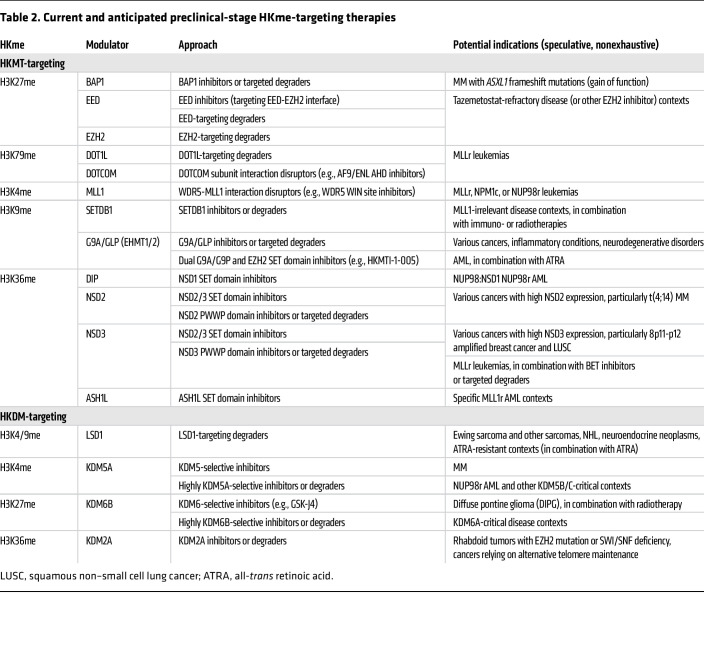
Current and anticipated preclinical-stage HKme-targeting therapies

**Table 1:**
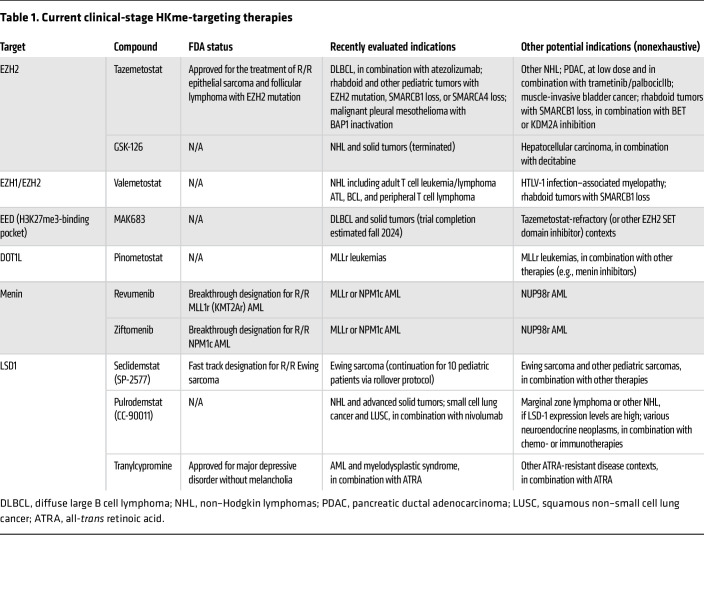
Current clinical-stage HKme-targeting therapies

## Version 1 10/15/2024

Electronic publication
